# Novel Phenobarbital-Loaded Nanostructured Lipid Carriers for Epilepsy Treatment: From QbD to *In Vivo* Evaluation

**DOI:** 10.3389/fchem.2022.908386

**Published:** 2022-08-17

**Authors:** Sebastian Scioli-Montoto, Maria Laura Sbaraglini, Jose Sebastian Cisneros, Cecilia Yamil Chain, Valeria Ferretti, Ignacio Esteban León, Vera Alejandra Alvarez, Guillermo Raul Castro, German Abel Islan, Alan Talevi, Maria Esperanza Ruiz

**Affiliations:** ^1^ Laboratory of Bioactive Compounds Research and Development, Department of Biological Sciences, School of Exact Sciences, National University of La Plata, La Plata, Argentina; ^2^ National Council for Scientific and Technical Research (CONICET), La Plata, Argentina; ^3^ Research Institute of Theoretical and Applied Physical Chemistry (INIFTA—CONICET—UNLP), Department of Chemistry, School of Exact Sciences, National University of La Plata, La Plata, Argentina; ^4^ Inorganic Chemistry Center (CEQUINOR—CONICET—UNLP), Department of Chemistry, School of Exact Sciences, National University of La Plata, La Plata, Argentina; ^5^ Physiopathology Chair, Biological Sciences Department, School of Exact Sciences, National University of La Plata, La Plata, Argentina; ^6^ Institute of Materials Science and Technology Research (INTEMA—CONICET—UNMdP), Mar del Plata, Argentina; ^7^ Nanomedicine Research Unit (Nanomed), Federal University of ABC (UFABC), Santo André, Brazil; ^8^ Max Planck Laboratory for Structural Biology, Chemistry and Molecular Biophysics of Rosario (MPLbioR, UNR-MPIbpC), Partner Laboratory of the Max Planck Institute for Biophysical Chemistry (MPIbpC, MPG), Center for Interdisciplinary Studies (CEI—CONICET), National University of Rosario, Rosario, Argentina; ^9^ Nanobiomaterials Laboratory, Center for Research and Development of Industrial Fermentations (CINDEFI—CONICET—UNLP), School of Exact Sciences, National University of La Plata, La Plata, Argentina

**Keywords:** phenobarbital, drug delivery, PTZ test, solid lipid nanoparticles (SLNs), nanostructured lipid carrier (NLC), epilepsy, anticonvulsant, release kinetic

## Abstract

Pharmacological treatments of central nervous system diseases are always challenging due to the restrictions imposed by the blood–brain barrier: while some drugs can effectively cross it, many others, some antiepileptic drugs among them, display permeability issues to reach the site of action and exert their pharmacological effects. The development of last-generation therapeutic nanosystems capable of enhancing drug biodistribution has gained ground in the past few years. Lipid-based nanoparticles are promising systems aimed to improve or facilitate the passage of drugs through biological barriers, which have demonstrated their effectiveness in various therapeutic fields, without signs of associated toxicity. In the present work, nanostructured lipid carriers (NLCs) containing the antiepileptic drug phenobarbital were designed and optimized by a quality by design approach (QbD). The optimized formulation was characterized by its entrapment efficiency, particle size, polydispersity index, and Z potential. Thermal properties were analyzed by DSC and TGA, and morphology and crystal properties were analyzed by AFM, TEM, and XRD. Drug localization and possible interactions between the drug and the formulation components were evaluated using FTIR. *In vitro* release kinetic, cytotoxicity on non-tumoral mouse fibroblasts L929, and *in vivo* anticonvulsant activity in an animal model of acute seizures were studied as well. The optimized formulation resulted in spherical particles with a mean size of ca. 178 nm and 98.2% of entrapment efficiency, physically stable for more than a month. Results obtained from the physicochemical and *in vitro* release characterization suggested that the drug was incorporated into the lipid matrix losing its crystalline structure after the synthesis process and was then released following a slower kinetic in comparison with the conventional immediate-release formulation. The NLC was non-toxic against the selected cell line and capable of delivering the drug to the site of action in an adequate amount and time for therapeutic effects, with no appreciable neurotoxicity. Therefore, the developed system represents a promising alternative for the treatment of one of the most prevalent neurological diseases, epilepsy.

## 1 Introduction

According to the World Health Organization (WHO), epilepsy is a non-transmittable chronic neurological disease that affects 50 million people globally, with a prevalence even higher than that of Parkinson’s disease (WHO, 2019). While the available battery of antiepileptic drugs (AEDs) currently manages to effectively treat almost 70% of epileptic patients, around 30%–40% turn out to be resilient to conventional therapies, suffering the so-called refractory epilepsy (Laxer et al., 2014).

Although this problem may be due to multiple factors, from the clinical point of view, one of the most studied hypotheses regarding drug-resistant epilepsy is the overexpression of efflux transporters at the blood-brain barrier (BBB) (Tang et al., 2017). The archetypical explanation is based on the experimental observation of high levels of expression of efflux transporters of the ABC family (ATP-binding cassette), such as P-glycoprotein (P-gp), at the BBB and/or at the epileptogenic focus. Accordingly, these transporters are responsible for reducing the amount of drug bioavailable at the site of action (Koehn, 2021).

In the past years, many efforts have been made for the development of new strategies intended to treat the drug-resistant phenotype, mainly focused on the discovery of new drugs that are not substrates for ABC transporters, e.g., P-gp (Couyoupetrou et al., 2016), as well as inhibitors of this efflux mechanism (Nanayakkara et al., 2018). An alternative strategy involves the development of nanometric carriers intended to transport the drug through the BBB, by-passing these efflux transporters. Synthetic polymer-based nanoparticles (NPs) are one of the most studied systems (Ren et al., 2019; Ahmad et al., 2020).

Among nanoparticulate vehicles, solid lipid NPs (SLNs) and nanostructured lipid carriers (NLCs) have gained ground in the field of drug delivery systems as they are considered non-toxic or of low risk, according to the nanotoxicological classification system proposed by Keck and Müller (2013). Generally, natural-origin lipid components are used as part of their matrices as well as surfactants that have been safely used in the pharmaceutical industry for years (Feeney et al., 2016). Whereas the lipidic phase of SLN is composed only of substances that are solid at room temperature, NLC allows the incorporation of a liquid lipid at room temperature (oil), that disrupts the structure of the lipid matrix, thus diminishing its reorganization at shelf conditions, which finally avoids the expulsion of the drug in early stages (Mehnert et al., 2001).

There are many examples of SLN developed for the treatment of diverse central nervous system (CNS) diseases in recent times (Scioli Montoto et al., 2020), from schizophrenia (Patel et al., 2019) to multiple sclerosis (Ojha and Kumar, 2018). In the particular case of epilepsy, there is still much to study, since, from the available battery of traditional AEDs, only carbamazepine (Nair et al., 2012; Scioli Montoto et al., 2018), oxcarbazepine (Scioli Montoto et al., 2021), and phenytoin (Motawea et al., 2018) have been loaded into SLN or NLC.

Phenobarbital (PB) is a first-line therapy for neonatal seizures (Kumar et al., 2021) that has been on the market for more than 100 years (Hauptmann and Alfred, 1912). Encapsulation of PB within novel pharmaceutical carriers is of interest due to several reasons. First, PB is a known P-gp substrate (Luna-Tortós et al., 2008), prone to active efflux from the brain. Therefore, its incorporation into nanocarriers might enhance its bioavailability in the CNS. Second, due to the number of years that it has been in the pharmaceutical market, it is an off-patent low-cost, accessible drug. This is a point to be noted since around 80% of the patients suffering from epilepsy live in low-to middle-income countries (Beghi et al., 2019). Accordingly, the development of novel, enhanced formulations of low-cost AEDs could be relevant for accessibility within low-income countries. Finally, it is worth mentioning that PB is a relatively lipophilic drug that may be readily incorporated into lipid nano-systems.

In the present study, a fractional factorial screening design for identifying the main factors affecting several properties of the final NPs was implemented. Then, a response surface methodology (RSM) by a central composite design (CCD) was used for the optimization of NLC containing PB. The optimized formulation was structurally characterized and tested *in vivo* in an animal model of acute seizures. The release mechanism, stability, and cytotoxicity were studied as well. Our results suggest this might be an alternative drug delivery system to be used in the treatment of epilepsies.

## 2 Materials and Methods

### 2.1 Materials

Lipid myristyl myristate (Crodamol® MM, melting point range = 36°C–40°C) and the oil (Crodamol^®^ GTCC-LQ, a triglyceride with saturated fatty acids, melting point = −4°C) were kindly donated by Croda (Argentina). Phenobarbital (99.9% as the basis) and Poloxamer 188 (Kolliphor^®^) were purchased from Saporiti (Argentina) and Sigma-Aldrich (Argentina), respectively. Other reagents used were of HPLC or analytical grade.

### 2.2 Preparation of Phenobarbital-Loaded Nanostructured Lipid Carriers

NLCs containing PB were prepared using the melt-emulsification by ultrasonication technique (Rodenak-Kladniew et al., 2019). The process started by melting myristyl myristate into a water bath at 70°C and then adding an adequate amount of oil (liquid lipid). Next, 50 mg of PB, as powder, was incorporated into the mixture. After 15 min, 20 ml of an aqueous solution of Poloxamer 188 (preheated at 70°C) was poured over the lipid phase. The resulting mixture was then sonicated using an ultrasonic processor (130W, Cole-Parmer, United States) equipped with a 6 mm titanium tip. Once the process was complete, it was allowed to cool down at room temperature. The amount of each ingredient, as well as the sonication time and frequency values, are presented in Table 1 **.**

**TABLE 1:** Factors (X), levels, and measured variables (Y) corresponding to the fractional factorial or screening design: formulations were synthesized according to the created design. Independent variables considered were: X_1_: the amount of solid lipid (mg); X_2_: amount of surfactant (mg); X_3_: amount of liquid lipid (μL); X_4_: time of sonication (min), and; X_5_: sonication power (%). Dependent variables were: Y_1_: particle size (nm); Y_2_: PDI, and; Y_3_: Z-potential (mV).

Formulation N°	Factors					Responses		
	X_1_	X_2_	X_3_	X_4_	X_5_	Y_1_	Y_2_	Y_3_
1	100	400	10	10	60	150,2	0,236	−9,56
2	100	400	10	40	60	144,0	0,233	−8,53
3	100	400	200	10	80	198,5	0,189	−11,4
4	100	400	200	40	80	201,2	0,118	−5,72
5	100	800	10	10	80	189,1	0,234	−4,42
6	100	800	10	40	80	188,5	0,212	−1,15
7	100	800	200	10	60	199,5	0,148	−5,45
8	100	800	200	40	60	174,2	0,165	−4,86
9	400	400	10	10	80	240,1	0,184	−14,1
10	400	400	10	40	80	242,6	0,114	−14,5
11	400	400	200	10	60	216,2	0,064	−18,1
12	400	400	200	40	60	245,8	0,158	−16,8
13	400	800	10	10	60	215,7	0,207	−11,2
14	400	800	10	40	60	208,6	0,200	−7,08
15	400	800	200	10	80	210,7	0,174	−4,49
16	400	800	200	40	80	191,4	0,208	−1,49

### 2.3 Fractional Factorial Screening Design

For the preliminary screening of relevant factors, a fractional factorial design of resolution V was employed (Cacicedo et al., 2019). A total of *k* = 5 independent factors ( $2_{V}^{k-1}$ ) of the NPs’ synthesis process were considered, at two levels each, to identify the main variables that affect the formulation size, size dispersion, and superficial charge. Therefore, the selected factors and dependent variables were: the amount of lipid (mg, X_1_), amount of surfactant (mg, X_2_), amount of oil (µl, X_3_), time of sonication (min, X_4_), sonication power (%, X_5_), particle size (Y_1_, nm), polydispersity index (PDI, Y_2_), and zeta potential (Z-potential, Y_3,_ mV). Factors, levels, and measured variables are summarized in Table 1. Package “FrF2” (Grömping, 2014) for R language (R Core Team, 2020) and R Studio (RStudio Team, 2018) were used for the creation and randomization of the design.

### 2.4 Optimization by the Response Surface Methodology Through Central Composite Design

A RSM through a CCD was used for the statistical optimization of the factors identified as relevant in the previous section, which were the amount of solid lipid and the amount of surfactant (X_1_ and X_2_, respectively). According to a rotatable CCD of two factors, each factor was tested at five different levels, and five replications of the center point were included (the final design and levels can be seen in Table 2). “RSM” package was used for the creation, randomization, statistical analysis, and plotting of the response surfaces (Lenth, 2009), and the “desirability” package was used for the joint optimization (minimization of all responses) (Kuhn, 2016).

**TABLE 2:** Factors (X), levels, and measured variables (Y) corresponding to the central composite or optimization design: formulations were synthesized according to the created design. Independent variables considered were: X_1_: the amount of solid lipid (mg) and X_2_: amount of surfactant (mg). Dependent variables were: Y_1_: particle size (nm); Y_2_: PDI, and Y_3_: Z-potential (mV). All the formulations contained 50 mg of PB.

Run	X_1_	X_2_	Y_1_	Y_2_	Y_3_
1	500.0	700.0	144.2	0.254	−10.3
2	500.0	700.0	145.6	0.248	−8.31
3	500.0	700.0	146.0	0.260	−8.95
4	250.0	900.0	183.3	0.376	−2.14
5	500.0	700.0	153.9	0.256	−9.35
6	750.0	500.0	199.2	0.240	−8.53
7	250.0	500.0	170.8	0.370	−1.10
8	853.6	700.0	164.1	0.263	−9.22
9	500.0	417.2	164.5	0.305	−11.2
10	750.0	900.0	169.7	0.238	−4.76
11	500.0	700.0	153.3	0.280	−5.12
12	500.0	982.8	155.4	0.282	−7.43
13	146.4	700.0	219.2	0.433	−2.74

### 2.5 Quantification of Phenobarbital

The chromatographic analysis of the samples was performed by high performance liquid chromatography (HPLC) in a Dionex Ultimate 3000 equipment (Thermo Scientific, Sunnyvale, CA, United States) with a dual gradient pump (DGP-3000) and a diode array detector (DAD-3000). A Hibar^®^ RP-18 (125 mm × 4 mm, 5 μm, Purosphere^®^ STAR, Merck KGaA, Darmstadt, Germany) column was used as the stationary phase, while the mobile phase consisted of a pH 7.0 KH_2_PO_4_ buffer: methanol (60:40) mixture, at a flow rate of 1.0 ml/min. Detection was executed at 239 nm. Before their injection (20 μl, fixed loop), samples were diluted with mobile phase (if necessary) and centrifuged at 15000*g* for 5 min.

### 2.6 Particle Size Distribution, Z-Potential and Polydispersity Index

Dynamic light scattering (DLS) was used for the determination of particles' mean diameter and polydispersity index (PDI) using a Nano ZS Zetasizer (Malvern Instruments, Worcestershire, United Kingdom) at 25°C in polystyrene cuvettes with a path length of 10 mm. Z-potential was measured by Doppler anemometry using the same equipment herein mentioned. Measurements were performed by triplicate in 10 mm path length capillary cells, with ultrapure water (Milli-Q®, Millipore, MA, United States).

### 2.7 Entrapment Efficiency and Drug Loading Capacity

Theoretical entrapment efficiency (EE, %) and drug loading capacity (DL, %) of the optimized formulation were determined by measuring the concentration of the free drug (non-encapsulated) in the solution. Around 500 µl of the final suspension of NPs were placed in Microcon® centrifugal filters (cut-off 10 kDa, Merck Millipore, Billerica, MA, United States), and centrifuged at 10,000*g* for 10 min. The concentration of free PB was measured by HPLC in the filtrate. EE and DL capacity (%) were then calculated considering the initial amount of PB added and the amount of free PB as shown in the following equations:

$$EE\left(\%\right)=\frac{W_{0}-\left(C_{FR}\times V_{f}\right)}{W_{0}}\times 100,$$ (1)

$$DL\left(\%\right)=\frac{W_{0}-\left(C_{FR}\times V_{f}\right)}{Lipid\;mass\;\left(mg\right)}\times 100,$$ (2)

where *W*_*0*_ corresponds to the initial mass of drug added to the formulation (mg), *C*_*FR*_ to the free drug concentration in mg/ml, and *V*_*f*_ to the final volume (ml) of formulation, set at 20 ml.

### 2.8 Transmission Electron Microscopy

Images were obtained at the transmission electron microscopy (TEM) Service of the School of Veterinary Sciences of the National University of La Plata, using a Jeol-1200 EX II-TEM microscope (Jeol, Columbia, MD, United States). The samples were diluted with ultrapure water (Milli-Q^®^, MA, United States). Two dilutions (1:10 and 1:500) of the optimized formulation NLC-PB and one dilution (1:10) of the non-loaded NLC (NLC-vehicle) were observed. Ten microliters of each dilution were spread onto a Cu grid of 400 mesh. After incubation, the sample excess was removed with filter paper. For contrast enhancement, one drop of phosphotungstic acid was added to the grid and incubated at 25°C for 1 min before excess removal. Finally, the grid was dried at room temperature.

### 2.9 Atomic Force Microscopy

Samples were diluted 1:1,000 with ultrapure water (Milli-Q^®^, MA, United States) and dropped onto freshly cleaved mica plates, with the addition of 10 µl of 10 mM CaCl_2_ solution. After 15 min, plates were washed with ultrapure water, and the excess was removed with filter paper (Whatman, Germany). Images were obtained at room temperature by a Multimode-Nanoscope V (Veeco, Santa Barbara, CA, United States) from the Nanoscopy Laboratory from the Theoretical and Applied Physical Chemistry Research Institute (INIFTA, CONICET, La Plata, Argentina). The microscope was operated in tapping mode, with an etched silicon probe model RTesp-Bruker (cantilever resonance frequency: 300 kHz; force constant 42 N/m; tip radius 8–12 nm). Typical scan rates were carried out at 1 Hz.

### 2.10 Differential Scanning Calorimetry Analysis

Thermal analysis of PB (free drug), NLC-PB, NLC without the drug (NLC-vehicle), and individual formulation components was performed by Differential Scanning Calorimetry (DSC) (PerkinElmer INC., model Parys 1, Waltham, MA, United States) under an inert atmosphere of N_2_. The heating rate was set at 10°C/min in the range of −80°C–200°C. For all assays, standard aluminum sample cells were used and filled with 2.0–14.0 mg of dried samples. Changes in the crystallinity index (CI, %) of myristyl myristate, measured as a percentage related to raw material after the synthesis process, were determined according to the equation used in previous reports (Scioli Montoto et al., 2018), considering the melting heat of pure myristyl myristate (*ΔH*_*MM*_ = 241.91 *J/g*) and a 3.0% lipid concentration (*C*_*lipid*_ _*phase*_, %w/v). The CI (%) was calculated as follows:

$$CI\left(\%\right)=\frac{\Delta H_{NLC\;dispersion}\left[Jg\right]}{\Delta H_{MM}\left[Jg\right]\times C_{lipid\;phase}\left[\%\right]}\times 100,$$ (3)

where $\Delta H_{NLC\;dispersion}$ corresponds to the melting heat of myristyl myristate in NPs.

### 2.11 Thermogravimetric Analysis

Thermal stability of PB, NLC-PB, NLC-vehicle, and individual formulation components was assessed by thermogravimetric analysis (TGA), using a TGA Q500 apparatus (TA Instruments, New Castle, DE, United States). About 4.0–32.0 mg of each sample were accurately weighted in a platinum pan and measurements were conducted at a heating rate of 10°C/min under N_2_ atmosphere.

### 2.12 X-Ray Diffraction Analysis

Crystalline structure analysis of the developed NPs and the formulation components were carried out in a PANanalytics X’Pert PRO diffractometer, equipped with an X-ray source with CuKα radiation at 40 kV and 40 mA (PANalytical, Philips PW 1830, Netherlands). Diffraction spectra data were collected over the 2θ range of 0°–45°. with an acquisition time of 1 s/s at each step of 0.02°.

### 2.13 Fourier Transform Infrared Spectroscopy

Interactions between the encapsulated drug and the NPs’ matrix were studied using a JASCO FT/IR—4,200 spectrometer (Jasco Inc., Easton, MD, United States) with 256 scans number for background correction, a high-energy ceramic source, and a DLARGS detector. All samples were prepared in solid-phase containing 5% (w/w) KBr.

### 2.14 *In Vitro* Release Assay

The release behavior of PB from the lipid NPs was studied in 500 ml of a KH_2_PO_4_/NaOH (pH 7.4) buffer solution, prepared according to USP 42 specifications (The United States Pharmacopeia 42, 2019). A rotating paddle apparatus (Vision Classic 6, Hanson Research, Chatsworth, CA, United States) was employed for the construction of each release profile. Rotating speed and bath temperature were set at 75 rpm and 37.0°C ± 0.5°C, respectively.

Five milliliters of each sample (equivalent to 12.5 mg of PB) were placed in pre-hydrated dialysis membranes (MWCO 10 kDa, Merck Millipore, MA, United States) and submerged into each vessel. Two controls were used: PB tablets (Bayer® Luminal, containing 100 mg of PB), as an example of an immediate-release product, and 5 ml of a PB solution (containing 12.5 mg of PB), to study the diffusional limit imposed by the dialysis bag. Samples were taken at regular intervals for 24 h and immediately centrifuged at 10,000*xg* for 5 min. The amount of PB in the withdrawn samples was determined by HPLC. NLC samples were analyzed by sextuplicate and the controls by triplicate.

To assess whether the dissolution rate depends on the initial amount of PB in the NPs, a formulation containing an equivalent of 7.5 mg of PB (instead of 12.5 mg), and identical amounts of the other components was prepared. This formulation was subjected to the dissolution test described in this section, under the same conditions and also in sextuplicate.

### 2.15 Cytotoxicity of Nanostructured Lipid Carrier Formulation in Mammalian Cell Cultures

The optimized formulation (PB theoretical concentration of 2.5 mg/ml, equivalent to 10.8 mM; MW = 232.2 g/mol) and a PB stock solution of the same concentration were diluted in an adequate amount of Dulbecco’s Modified Eagle Medium (DMEM) to achieve the concentrations under study (1, 2, 3, and 4 mM of PB).

MTT [3-(4,5-dimethylthiazol-2-yl)-2,5-diphenyltetrazolium bromide] test was applied for the evaluation of PB and NLC-PB cytotoxicity on L929 non-tumoral mouse fibroblasts (Mosmann, 1983): a mitochondrial complex is responsible for catalyzing formazan crystals formation from soluble tetrazolium bromide, complex that is only active in viable cells, thus assessing mitochondrial activity. L929 ATCC CRL 6364^®^ cell line was grown in DMEM supplemented with 10% of Fetal Bovine serum, 100 U/ml of penicillin, and 100 μg/ml of streptomycin and maintained in a humified atmosphere containing 5% CO_2_ at 37°C. Cells were seeded in 96-well plates at 20,000 cells per well and incubated overnight to allow a correct attachment of cells. Cells were then treated with dilutions of the optimized formulation and PB solution (in the previously mentioned concentrations) at 37°C for 24 h. After this time, the supernatant was replaced with 100 µl of MTT (0.5 mg/ml), which was discarded after 3 h. Optical density of colored formazan was measured spectrophotometrically at 570 nm after cell treatment with DMSO.

### 2.16 Stability Studies

Stability studies of the optimized formulation were performed under storage conditions. Samples were kept at 2°C–8°C (refrigerator). Individual glass vials for each replicate and time were used. Analysis of particle size, PDI, and Z-potential was made using the procedures described above, immediately after the synthesis and after 45 days of storage.

### 2.17 Anticonvulsant Activity

The pharmacological activity *in vivo* was assessed following the standard procedures provided by the antiepileptic drug development (ADD) Program of the National Institute of Neurological and Communicative Disorders and Stroke (Kehne et al., 2017) and according to the guidelines of the Institutional Committee on Care and Use of Experimental Animals (CICUAL, School of Exact Sciences, National University of La Plata, Argentina). Swiss albino mice weighing between 18 and 25 g at the time of testing were provided by the School of Veterinary Sciences (UNLP). All mice were housed in colony cages with a maximum of 10 mice per cage with light cycles of 12 h light/darkness and controlled room temperature between 20°C and 22°C. *Ad libitum* food and water were provided. Adaptation of mice to the new environment was carried out for 5 days, injecting 0.1 ml of normal saline (NS), intraperitoneally (ip).

Seizures were elicited by ip injection of 10 ml/kg of Pentylenetetrazol (PTZ) at 25 mg/kg dose. One positive control (25 mg/kg PB solution), two negative controls (NS and NLC-vehicle), and nanoparticle suspension (NLC-PB, equivalent to 25 mg/kg) were injected. The anticonvulsant activity was tested at 0.25, 1.0, and 4.0 h post-PTZ administration. A total of five mice per treatment and per time point were used for this assay. A maximal volume of 10 ml/kg of freshly made solutions was ip administered. The PTZ dose administered produces clonic seizures that may last for a period of at least 5 s in 97 percent (CD97) of the rodents analyzed. After injection of PTZ, the animals are placed in isolation cages and observed for 30 min for the presence or absence of a seizure. The absence of clonic seizures, characterized by fore and/or hind legs, jaws, or vibrissae clonus spams for at least 3 s, is considered as protection against PTZ-induced seizures (National Institute of Neurological Disorders and Stroke, https://panache.ninds.nih.gov/TestDescription/TestPST).

Finally, RotoRod test was used to assess the possible neurotoxic effects of the injected formulations. Considering that a normal mouse is capable of maintaining its equilibrium on a rotating rod (6 rpm) for long periods, failure to maintain this equilibrium during 1 min was considered an indication of a possible neurological deficit.

### 2.18 Statistical Analysis

Experiments were carried out with a minimum of three independent replicas unless otherwise stated. For the statistical comparison of two or more groups, Student t-test or ANOVA (followed by Tukey’s HSD test if necessary) test was performed, respectively. A significance level of 0.05 was used. Prior to every parametric analysis, the assumptions of normality and homogeneity of variance were checked. Other statistical analyzes are described in the corresponding section.

## 3 Results

### 3.1 Main Factor Screening

In the present study, 16 different formulations were prepared according to the fractional factorial design previously described. Table 1 shows the different formulations that were synthesized according to such design, along with the values obtained for the dependent variables.

For the dependent variable particle size (Y_1_), the analysis of variance (ANOVA) showed that the amount of lipid (X_1_, mg) was statistically significant (*p* < 0.01), while the other factors were not (*p* > 0.05). In the case of PDI, none of the factors resulted statistically significant (*p* > 0.01). Finally, for Z-potential, ANOVA showed that both the amount of lipid (X_1_, mg) and the amount of surfactant (X_2_, mg) were statistically significant variables (*p* < 0.01). Therefore, X_1_ and X_2_ were selected for further optimization. The remaining variables, such as amount of liquid lipid (X_3_, μl), time of sonication (X_4_, min), and sonication frequency (X_5_, %), were set at 24 μl, 20 min, and 80%, respectively.

### 3.2 Optimization by the Response Surface Methodology Through Central Composite Design

A 2-factor, 5-levels CCD was applied to further optimize the formulations. Factors’ levels and the responses obtained for each run are shown in Table 2. Four different models were assayed for the generation of the response surfaces: 1) a multiple linear regression model (first-order); 2) a multiple linear regression model considering first-order interactions between factors; 3) a pure quadratic model, considering only first-order and quadratic terms, and; 4) a second-order model, with first-order, interactions and second-order terms.

**FIGURE 1:**
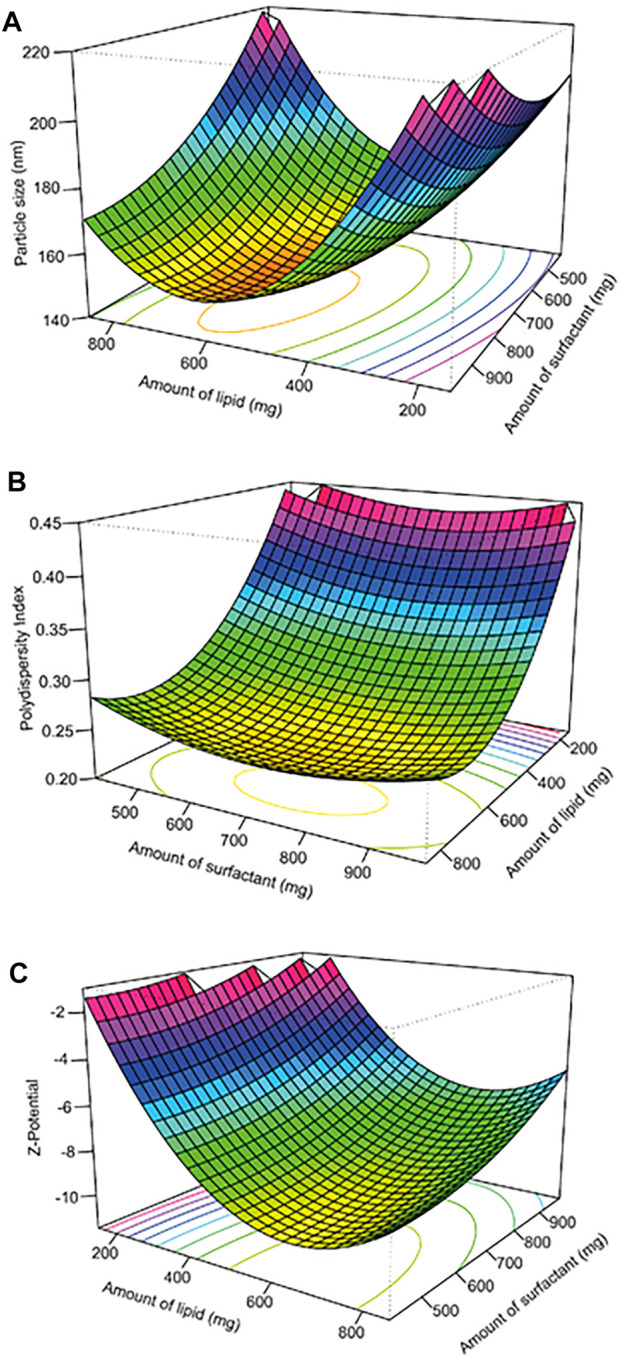
Response surfaces for particle size **(A)**, PDI **(B)**, and Z-potential **(C)** as a function of the amount of lipid (mg) and surfactant (mg).

Best fitting models for each response were: second-order for particle size (*R* ^*2*^ = 0.80) and pure quadratic for PDI (*R* ^*2*^ = 0.97) and Z-potential (*R* ^*2*^ = 0.60). Response surfaces are shown in Figure 1. Finally, a general desirability function using the “*desirability”* package was applied for the simultaneous minimization of all responses. The Nelder-Mead (Olsson and Nelson, 1975) approach was used for the determination of the optimal point.

The best value obtained for general desirability was 0.90 corresponding to 616.9 mg of lipid and 706.8 mg of surfactant. Thus, a new formulation was synthesized according to these results, in triplicate, and the responses were measured. The results, along with the prediction error and the relative standard deviation, are shown in Table 3 **.**

**TABLE 3:** Individual values, mean, prediction error (S_pred_), and relative standard deviation (RSD) were obtained for the particle size (nm), PDI and Z-potential (mV) of the optimized formulation.

Response	Values	Mean	S_pred_	RSD_pred_ (%)
Particle size (nm)	180,1	178.6	9.52	5.35
	175,0			
	180,6			
PDI	0,250	0.244	0.009	3.69
	0,230			
	0,252			
Z-potential (mV)	−12,6	−12.2	1.57	12.9
	−11,4			
	−12,5			

From here on, this optimized formulation will be called NLC-PB. The values of EE (%) and DL (%) (measured as described in Section 2.7) were 98.2% and 7.7%, respectively.

### 3.3 Physicochemical characterization by Atomic Force Microscopy, Transmission Electron Microscopy, Differential Scanning Calorimetry, Thermogravimetric Analysis, X-Ray Diffraction Analysis, and Fourier Transform Infrared Spectroscopy Analysis

In addition to the measurement of the particle size obtained by DLS, the Atomic Force Microscopy (AFM) technique was used to study the particles’ morphology. In the particular case of lipid NPs, AFM observation is considered a complementary method, particularly useful for observing the shape of the particles (Peixoto et al., 2005). On the other hand, the technique is less relevant for the quantification of dimensions, since particles’ deformation usually occurs (Dubes et al., 2003; Mazuryk et al., 2016).

**FIGURE 2:**
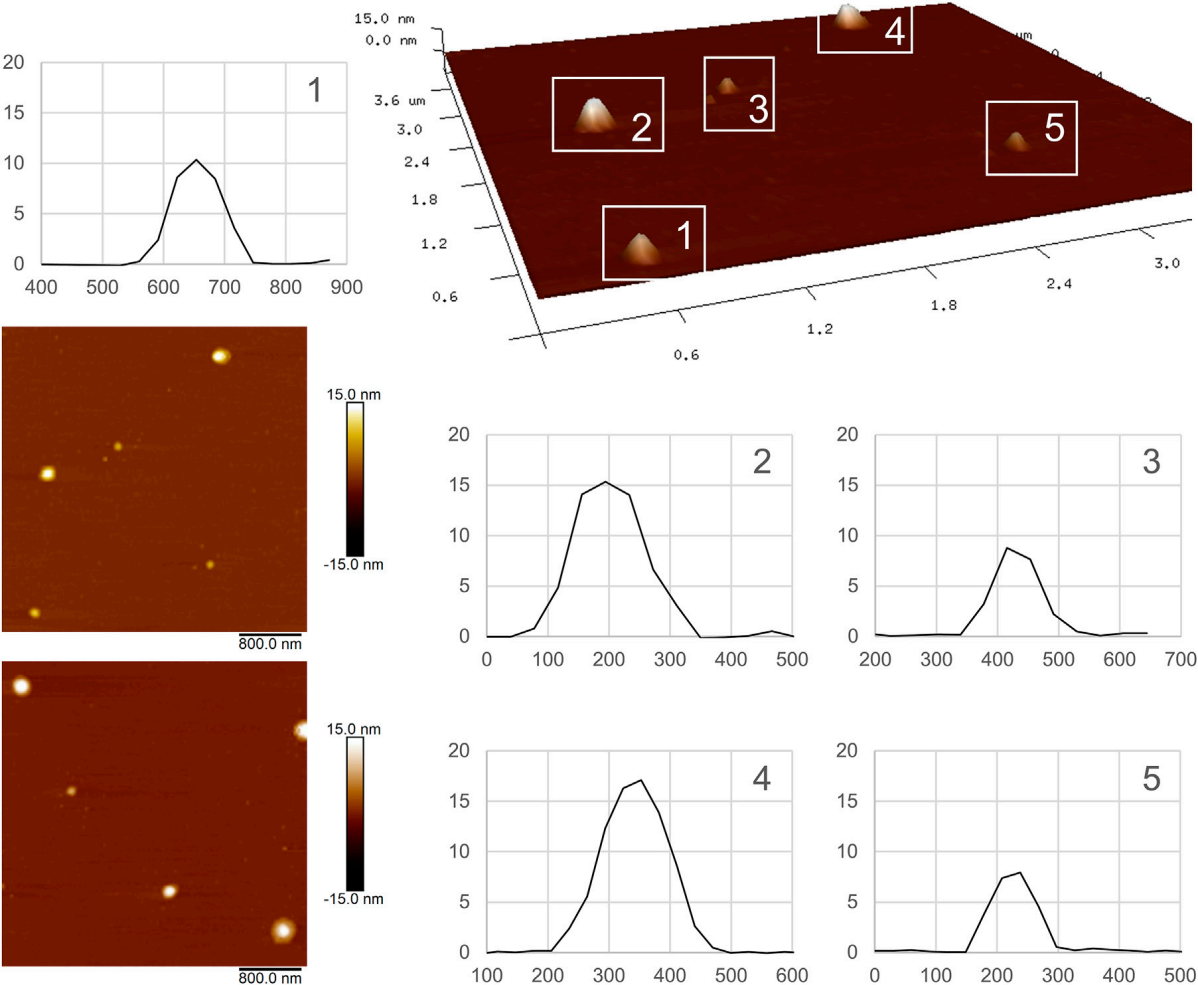
AFM images of NLC-PB. On the left, two independent 2D images are presented. The 3D image corresponds to the upper one. In the size curves, the *X*-axis has an arbitrary origin, and thus it serves to measure differences (diameters). Attractive forces with the support and weak forces of the probe generate flattening in the analyzed NPs resulting in smaller z particle size.

Figure 2 shows two independent 2D images obtained by this methodology (left), the 3D projection of the upper one and the particles’ size curves. As it was expected, the diameters obtained by this technique turned out to be of the same order but slightly greater than the value reported by DLS. In the *xy* plane, sizes of the order of 200 nm could be observed, while in the *z*-axis the size was around 10–15 nm.

**FIGURE 3:**
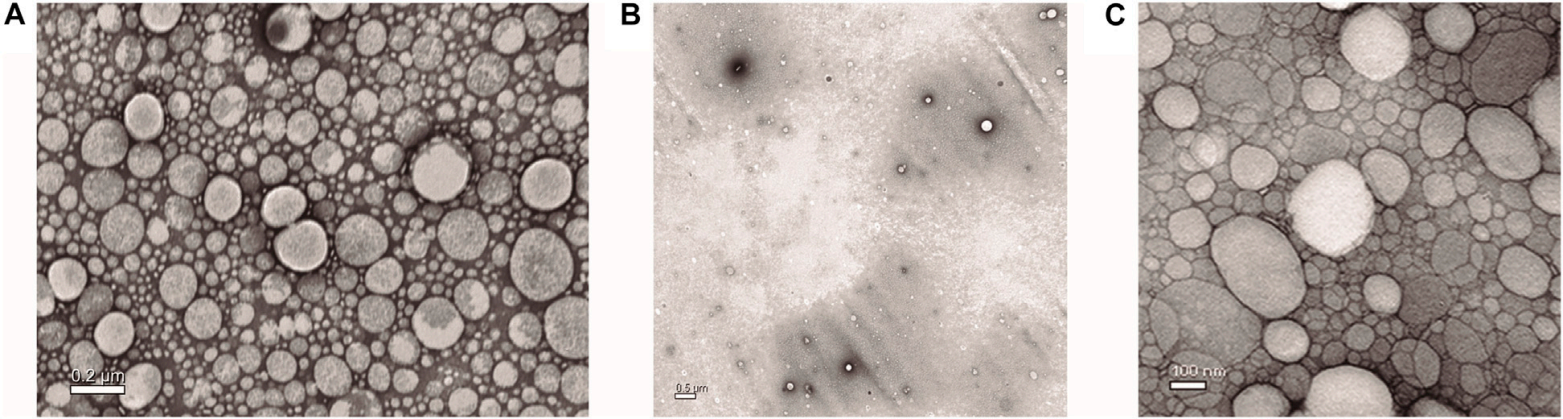
TEM images of: **(A)** 1:10 dilution, **(B)** 1:500 dilution of NLC-PB optimized formulation, and **(C)** 1:10 dilution of empty NLC (NLC-vehicle).

TEM images of 1:10 and 1:500 dilutions of NLC-PB, and a 1:10 dilution of empty NLC (NLC-vehicle) can be seen in Figures 3A–C, respectively. These images confirm that the particles possess a spherical shape with a mean particle size of 185 ± 28 nm, a result obtained with the ImageJ Software (Schneider et al., 2012). These results confirmed those obtained by DLS and AFM.

**FIGURE 4:**
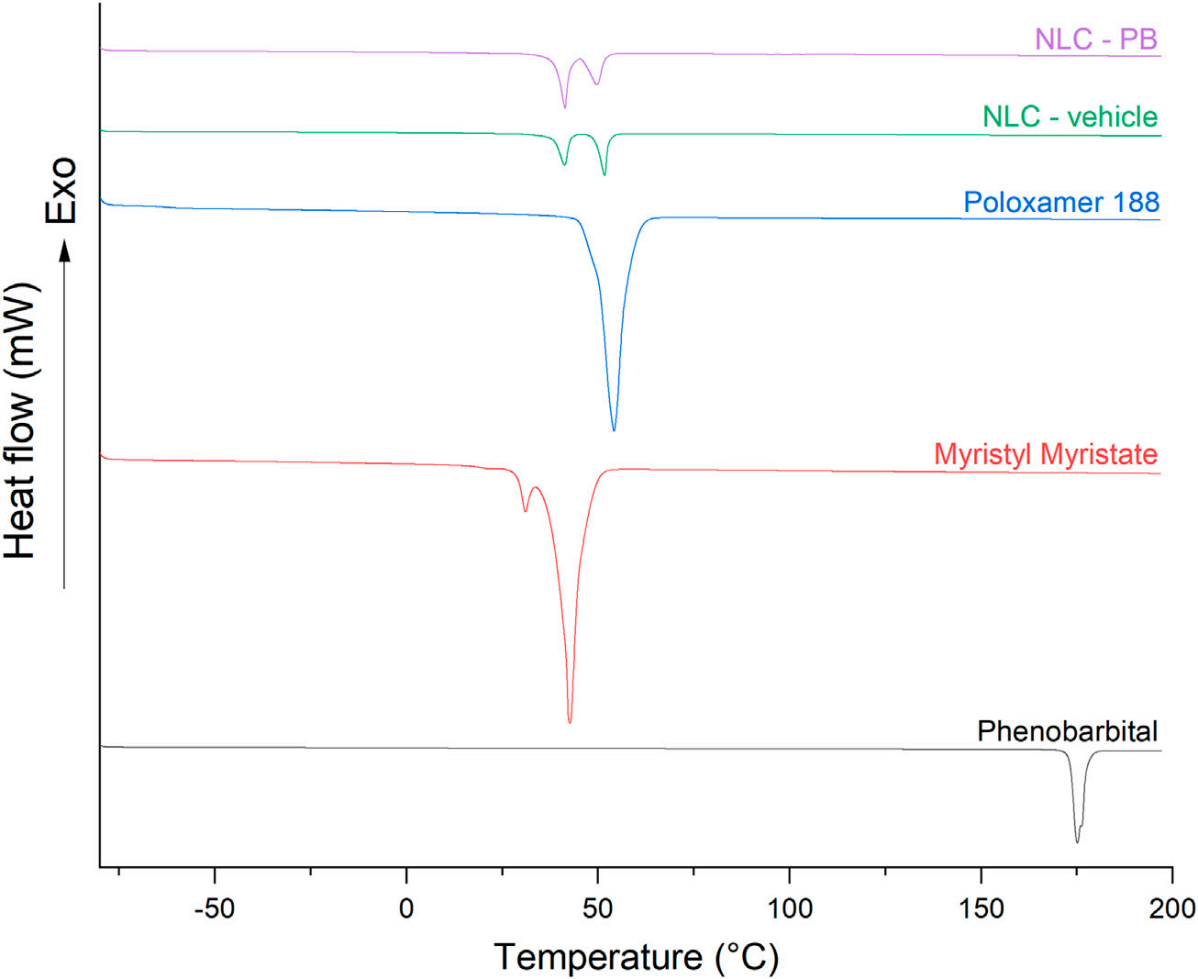
Stacked DSC thermograms corresponding to PB, myristyl myristate, Poloxamer 188, NLC-vehicle, and NLC-PB. Note: *Y*-axis scale is the same for all thermograms.

Individual components (drug and excipients) and formulations (with and without PB) were thermally characterized by DSC and TGA. Figure 4 shows the DSC thermograms of PB, myristyl myristate, Poloxamer 188, NLC-vehicle (without PB), and NLC-PB. DSC curve of PB showed an endothermic peak at 175.4°C with a melting heat of 127.1 J/g, which corresponds to polymorph I of PB (Zencirci et al., 2010). Myristyl myristate showed two endothermic peaks (at 31.1°C and 42.8°C, with a total melting heat of 241.6 J/g) corresponding to the melting processes of the different types of polymorphs that lipids may present: the *α* polymorph is a metastable structure with the lowest crystal lattice and, consequently, the lowest melting point. The polymorph β’ is an intermediate-stability polymorph (ten Grotenhuis et al., 1999). In turn, the curve corresponding to Poloxamer 188 showed an endothermic peak at 54.2°C with a melting heat of 156.6 J/g. Concerning the NPs, NLC-vehicle and NLC-PB showed only two endothermic peaks at 41.3°C and 51.7°C, the first one corresponding to the melting point of myristyl myristate and the second one to Poloxamer 188. At the same time, NLC-PB thermogram showed no peak that could be associated with the melting process of PB. The shift to a lower melting point of the lipid polymorph in the formulation (with respect to the raw material) is indicative of a decrease in the ordering of its crystal lattice (Freitas and Müller, 1999).

Table 4 shows the values of melting points, melting heat, and crystallinity index (calculated according to Eq. 3) of the aforementioned samples. The crystallinity index (%) of raw materials and NPs were calculated taking as reference the melting peak of myristyl myristate.

**TABLE 4:** Melting points (T_m_), melting heat (ΔH_m_), and crystallinity index (CI) of raw materials and NPs with and without PB. Crystallinity indexes of NLC-vehicle and NLC-PB were calculated according to Eq. 3.

Sample	T_m_ (°C)	ΔH_m_ (J/g)	CI (%)
Phenobarbital	175.4	127.1	100
Myristyl myristate	31.1–42.8	241.6	100
Poloxamer 188	54.2	156.6	100
NLC-vehicle	41.3–51.7	91.6	12.6
NLC-PB	41.4–49.7	96.7	13.3

**FIGURE 5:**
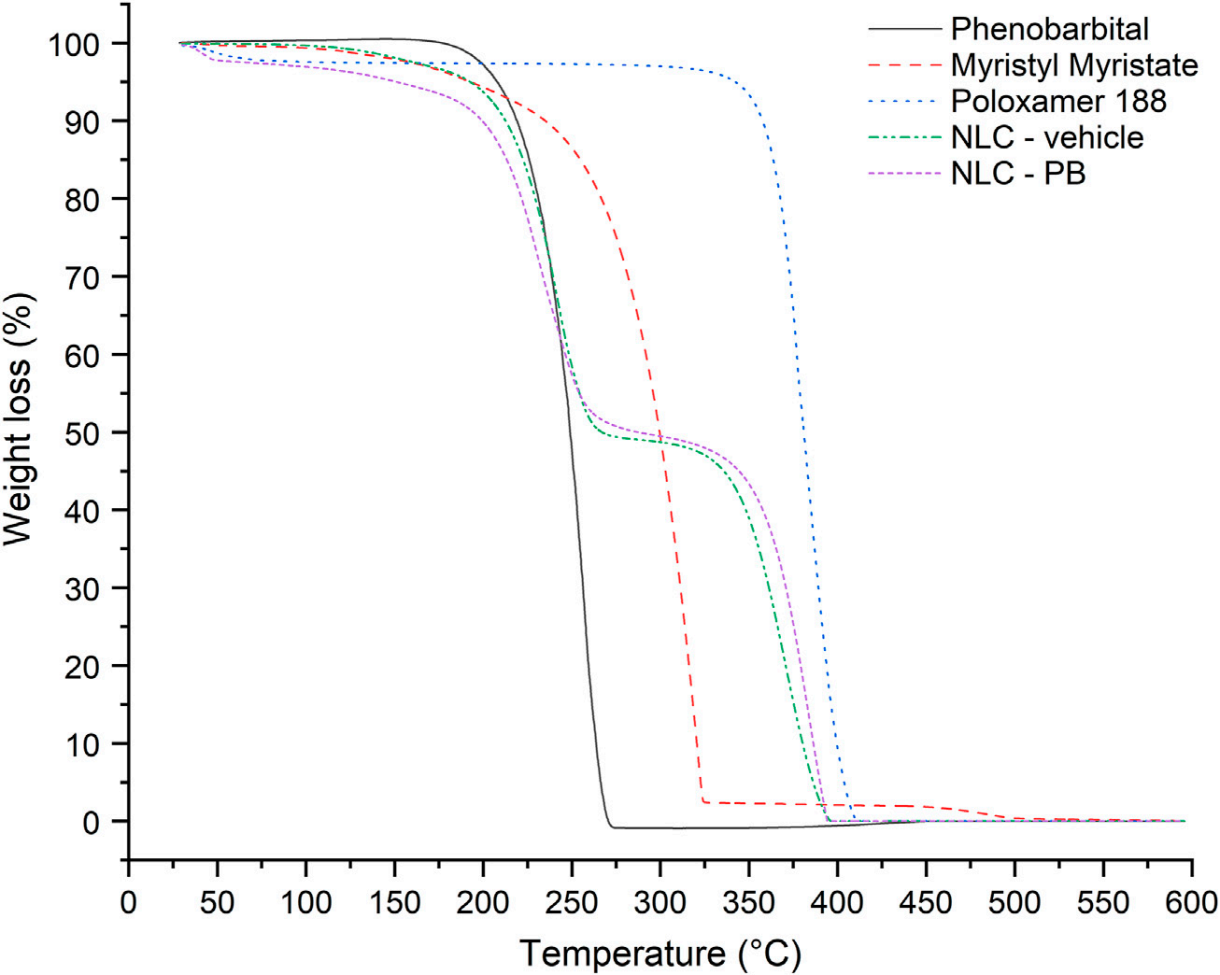
TGA thermograms of PB, myristyl myristate, Poloxamer 188, NLC-vehicle, and NLC-PB were obtained in the range of 30°C–600°C.

TGA was performed to study possible changes in the thermal properties of NPs due to the incorporation of PB (Figure 5). Thermal decomposition of PB proceeded in one step of mass loss (100%), between 160°C and 275°C. On the other hand, myristyl myristate showed an initial mass loss of 5% up to 190°C, temperature at which the main mass loss step begins (95%), ending at 323°C. Poloxamer 188 showed a single step of mass loss starting at 314°C and ending at 414°C. Finally, both NLC-vehicle and NLC-PB displayed mass losses in two steps: the first one between 180°C and 260°C (47.8%) and the second one between 260°C and 410°C (49.9%).

**FIGURE 6:**
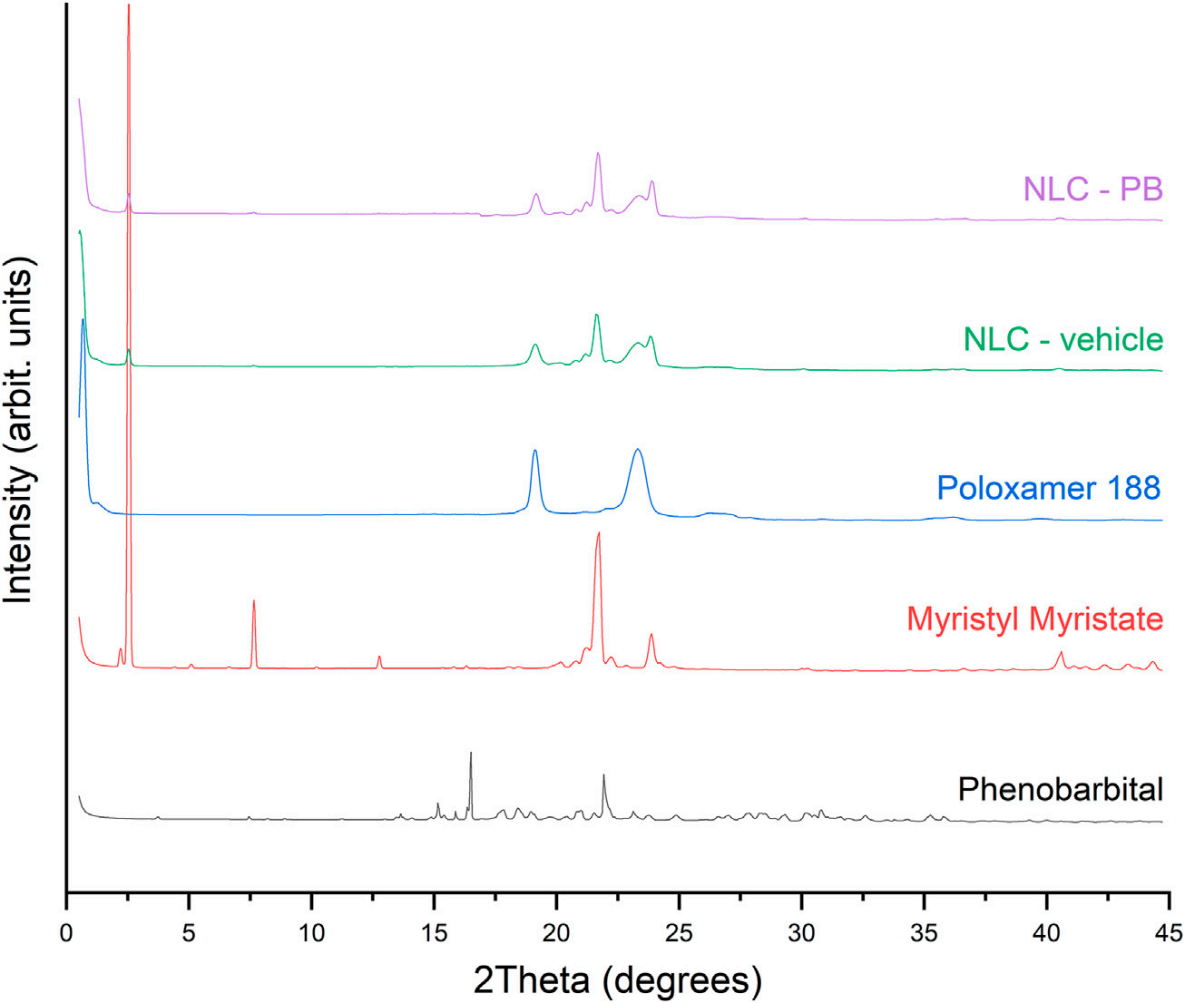
XRD patterns of raw materials and nanoparticles with and without phenobarbital. Note: *Y*-axis scale is the same for all x-ray diffraction patterns.

The crystalline structure of raw materials and NPs with and without PB was analyzed by X-Ray Diffraction Analysis (XRD). Diffraction profiles obtained can be seen in Figure 6, and the exact peak positions and *d*-spacings are presented in Table 5 **.** The three main peaks presented by PB, along with the information obtained by DSC, suggest that the pure drug corresponds to its polymorphic form I (Zencirci et al., 2010). On the other hand, the main signals of myristyl myristate and their corresponding *d*-spacings are characteristic of *α* and *β*’ polymorphs of lipid compounds (Jenning et al., 2000). Concerning to Poloxamer 188, it showed two main peaks at 19.13° and 23.29°. Both the NLC-vehicle and NLC-PB showed four main peaks at 19.18°, 21.64°, 23.33°, and 23.90°, the last two being partially overlapped. As it can be seen in Figure 6, none of the main peaks of PB could be observed in the diffraction pattern of the optimized formulation (purple curve).

**TABLE 5:** Peaks position and *d-*spacings of raw materials and NPs with and without PB were measured by XRD at *λ* = 0.154 nm.

Sample	Peak position (2θ)	Angle (*θ*)	*d-*spacing (Å)
Myristyl myristate	21.67	10.8	0.41
	23.87	11.9	0.37
Poloxamer 188	19.13	9.56	0.46
	23.29	11.6	0.38
Phenobarbital	15.17	7.58	0.58
	16.50	8.25	0.54
	21.96	11.0	0.40
NLC-vehicle	19.13	9.56	0.46
	21.64	10.8	0.41
	23.33	11.7	0.38
	23.90	11.9	0.37
NLC-PB	19.18	9.59	0.46
	21.64	10.8	0.41
	23.33	11.7	0.38
	23.90	11.9	0.37

**FIGURE 7:**
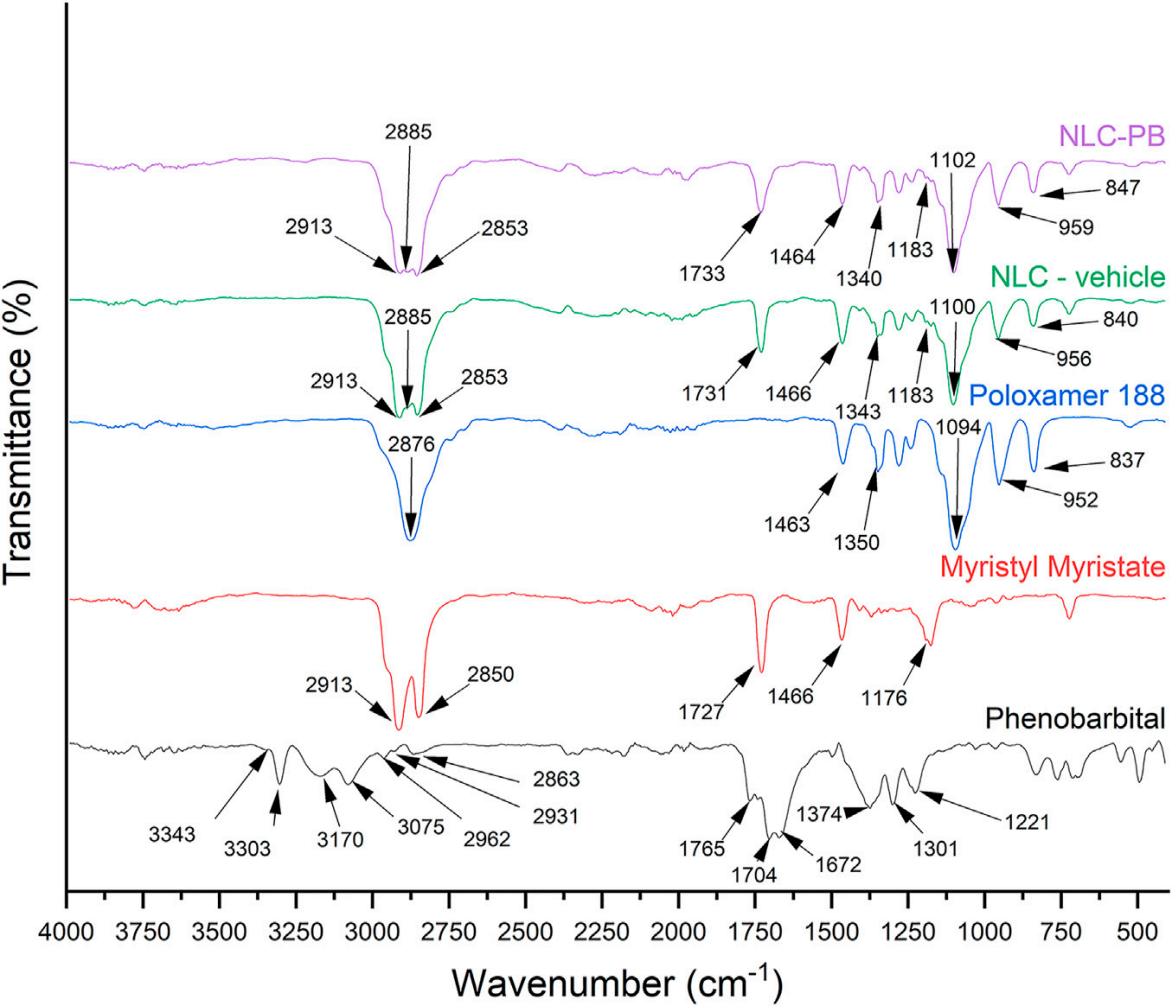
Overlaid FTIR spectra of PB, myristyl myristate, Poloxamer 188, NLC-vehicle, and NLC-PB.

Possible interactions among different formulations’ components were analyzed by Fourier Transform Infrared Spectroscopy (FTIR). Figure 7 shows overlaid spectrums of raw components, NLC-PB and NLC-vehicle. The spectrum of PB showed the characteristics bands of its molecular structure: a low-intensity band at 3,343 cm^−1^ corresponding to the overtone associated with the stretching of C=O bonded to the carbon that possesses phenyl and ethyl substituents. At frequencies of 3,303 cm^−1^ and 3,075 cm^−1^ there could be seen bands associated with the symmetric and asymmetric stretching of N-H groups, respectively. Bands at 3,170 cm^−1^, 2,962 cm^−1^, 2,931 cm^−1^, and 2,863 cm^−1^ could be assigned to the stretching of CH_2_ and CH_3_ groups. Over lower frequencies, bands corresponding to the stretching of C=O groups could be observed: the band located at 1765 cm^−1^ could be assigned to the stretching of C=O groups located between N-H groups (-NH-CO-NH-) and the band at 1,672 cm^−1^ to the stretching of C=O groups at the same plane as N-H groups (-C-CO-NH-). Lastly, there can be seen bands at 1,374, 1,301, and 1,221 cm^−1^ attributable to the stretching frequencies of C-N bonds (Zencirci et al., 2010).

Myristyl myristate showed two characteristic bands at 2,913 cm^−1^ and 2,850 cm^−1^ associated with symmetric and asymmetric stretching of the carbon chains’ methyl and methylene groups, respectively. The bands located at 1,727 cm^−1^ and 1,176 cm^−1^ could be assigned to the stretching of C=O groups and C-O bond, respectively. Lastly, bands located at 1,466 cm^−1^, 722 cm^−1^, and 708 cm^−1^ correspond to the bending movement of methyl and methylene groups (first band) (Islan et al., 2016; Nosal et al., 2020) and rocking movement characteristic of parallel orthorhombic packing present in lipid polymorph β’ (de Ruig, 1971).

Poloxamer 188 spectrum showed a characteristic band due to the stretching of O-H groups at 3,520 cm^−1^. At higher frequencies, bands at 2,876 cm^−1^ and 1,463 cm^−1^, corresponding to the symmetric stretching of C-H bonds of the copolymer aliphatic chains and the bending of -CH_2_- groups, were observed. In addition, because of the bending of O-H groups and the symmetric stretching of C-O-C bonds, bands at 1,350 cm^−1^ and 1,094 cm^−1^, respectively, could be observed. Lastly, two bands can be observed at 952 cm^−1^, and 837 cm^−1^ that can be associated with the symmetric and asymmetric stretching of C-C-O bonds, respectively (Li et al., 2016).

As can be seen in Figure 7, both NLC-vehicle and NLC-PB spectrum clearly show the contribution of myristyl myristate and Poloxamer 188, with characteristics bands that showed small shifts maybe attributable to weak interactions after NPs’ formation. On the other hand, characteristic bands of PB were not observed in the NLC-PB spectrum.

### 3.4 *In Vitro* Release

**FIGURE 8:**
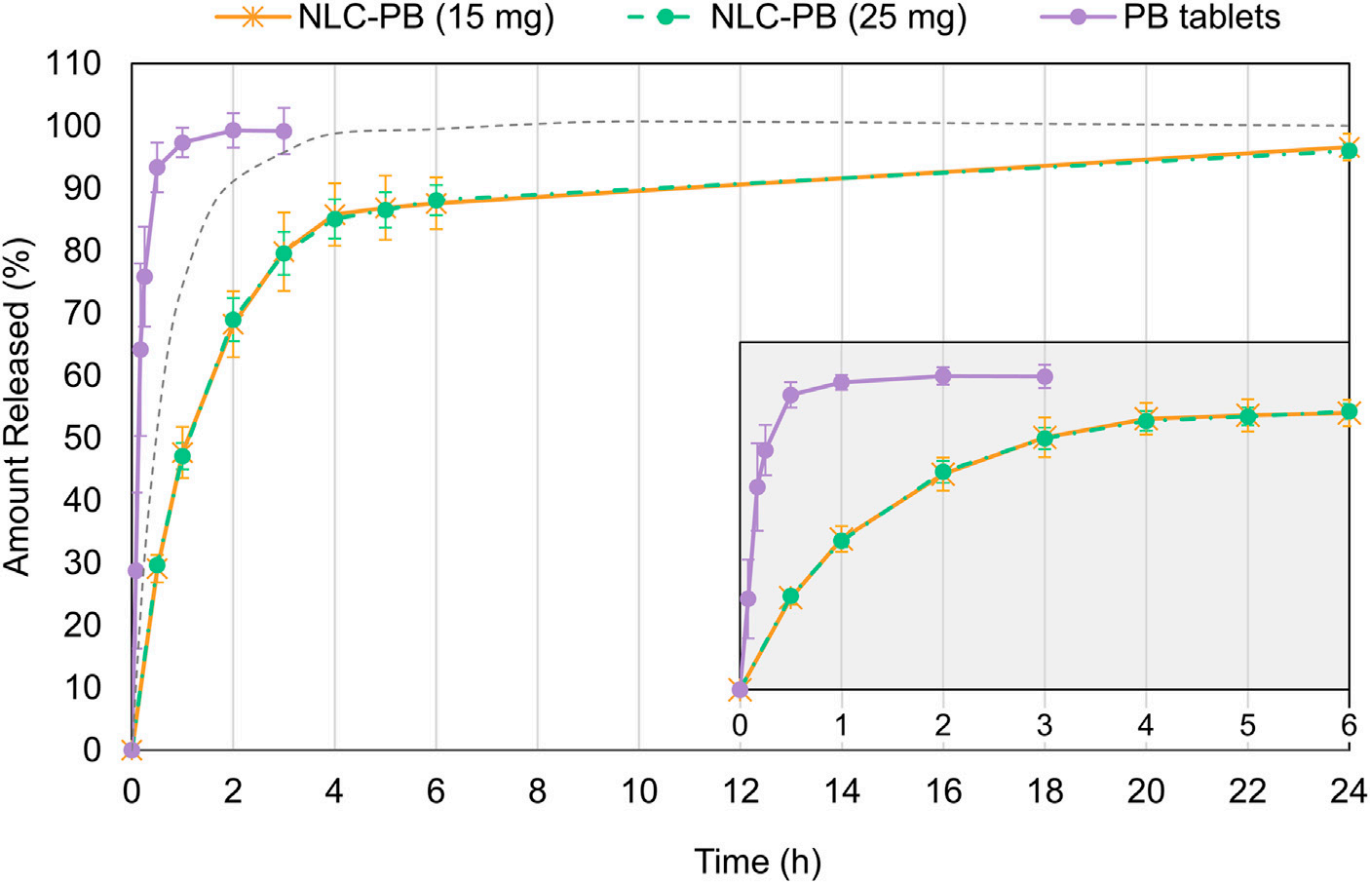
Release profiles of PB from NLC with different drug loading. Two controls were included: immediate-release PB tablets (Bayer Luminal®, 100 mg) and a solution of PB inside the dialysis bag (discontinuous grey line). The inserted graph corresponds to the time range from 0 to 6 h. Initial point (*t* = 0) was included for graphical purposes.

Figure 8 shows the release profiles of the NLC-PB formulations, with two different drug loads, together with the profile of the controls (PB immediate-release tablets, and a solution of PB inside the dialysis bag). From Figure 8, it can be observed that the release rate was not limited by the dialysis bag and that both formulations exhibited a slower release than the immediate-release formulation, with differences ranging from 60% (at 0.5 h) to 30% (at 3 h). On the other hand, an almost perfect overlap was observed between the profiles corresponding to the NLC-PB with different PB amounts, which is consistent with a release mechanism driven by the diffusion or desorption of the drug from the matrix (Baker and Lonsdale, 1975).

Therefore, experimental data were fitted to a mathematical model developed for monolithic systems, where the drug is intimately mixed with the components that limit its release (the NLC matrix, in this case). For the modeling of release profiles, this diffusion model (the exact expression of which was originally derived by Crank (Crank, 1975)) is applied under the form of two approximate expressions, depending on whether the initial portion (dissolved fraction up to 40%) or final portion (from 60% dissolved) of the curve is considered (Baker and Lonsdale, 1975). The corresponding expressions, for systems with spherical geometry, are

MtM∞=6(Ktπ)12−3Kt                         for    0≤MtM∞≤0.4, (4)

$$\frac{M_{t}}{M_{\infty }}=1-\frac{6}{\pi^{2}}\;exp\left(\pi^{2}Kt\right)\;\;\;\;\;\;\;\;\;\;\;\;\;\;\;\;\;\;\;\;for\;\;\;\;\;0.6\leq \frac{M_{t}}{M_{\infty }},$$ (5)

where $M_{t}$ is the mass released at time *t*, $M_{\infty }$ is the total drug content (initial load) and *K* is a constant related to the shape and the material of the release system.

The early time approximation predicts a linear relationship between the dissolved fraction and the 0.43 power of time, a result obtained by Ritger and Peppas (1987) when postulating their release model based on Eq. 4, also known as the power law. However, and because a fractional release above 40% was already achieved at 1 h, the late time expression (Eq. 5) was used here, applied to time points starting from 2 h (i.e., when the release was above 60%). By doing so, a value of *K* = 0.033 h^−1^ was obtained, and with it, the predicted values for the initial times were calculated according to Eq. 4. Finally, a latency time (lag time) was incorporated as an additional parameter of the model, to take into account the effect of the dialysis membrane used during the experiment (Fotaki et al., 2014; Fugit and Anderson, 2014). The predicted lag time was 0.2 h, and the final model exhibited a good fit to the data, with *R* ^*2*^ and MSE (mean squared error) values of 0.960 and 0.002, respectively. Infostat software (National University of Córdoba, Córdoba, Argentina) was used for the fitting, and the data of both formulations (7.5 and 12.5 mg) were grouped.

### 3.5 Cytotoxicity of Nanostructured Lipid Carrier Formulation in Human Cell Culture

**FIGURE 9:**
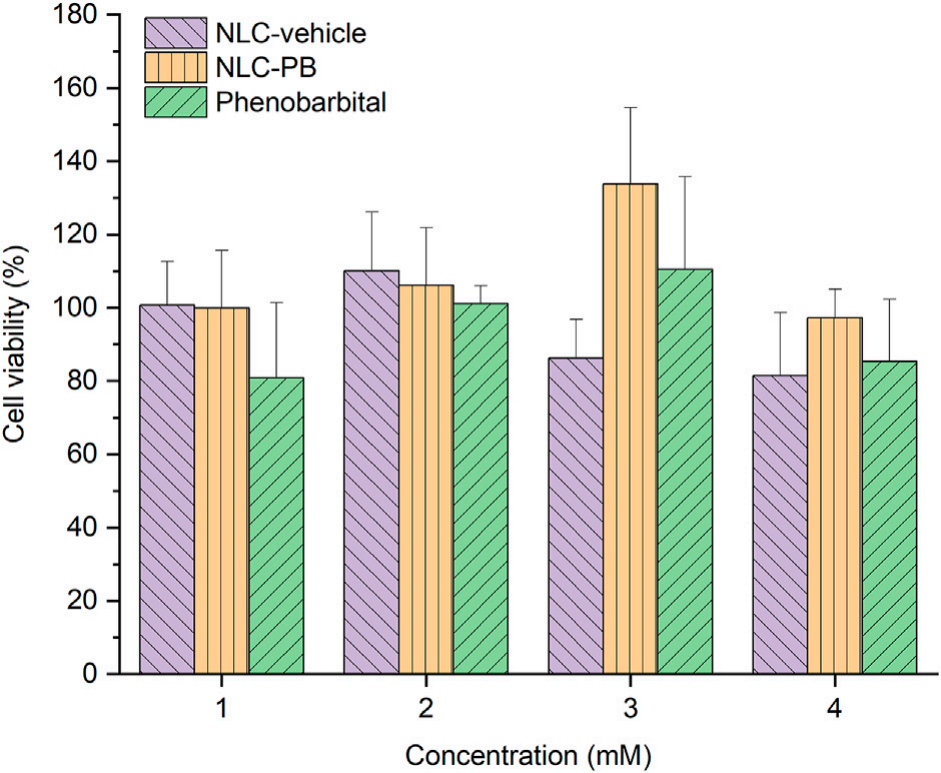
Cell viability analysis by MTT assay. L929 cells were treated with different concentrations (1–4 mM) of NLC-PB, NLC-vehicle, and PB.

The toxicity of NLC-PB, NLC-vehicle, and PB was evaluated by a cell viability assay using L929 cells by the MTT method. L929 cells were selected as they are commonly used to evaluate the *in vitro* cytotoxicity of different types of NPs (Bueloni et al., 2020). As can be seen in Figure 9, the assayed formulations were non-toxic for L929 cells over the concentration range from 1 to 4 mM, and no statistically significant differences were found among the treatments (two-way ANOVA, *α* = 0.05).

### 3.6 Stability Studies

Stability studies were carried out for 45 days, with three independent replicas. After 45 days of storage, particle size was 180.2 ± 0.7 nm, PDI was 0.199 ± 0.001 and Z-potential was −10.2 ± 0.4 mV. Student t-test did not show statistically significant differences with regard to t = 0, for any of the three variables (*p*-values were 0.709, 0.874, and 0.354 for particle size, PDI, and Z-potential, respectively).

### 3.7 Anticonvulsant Activity Assay

**FIGURE 10:**
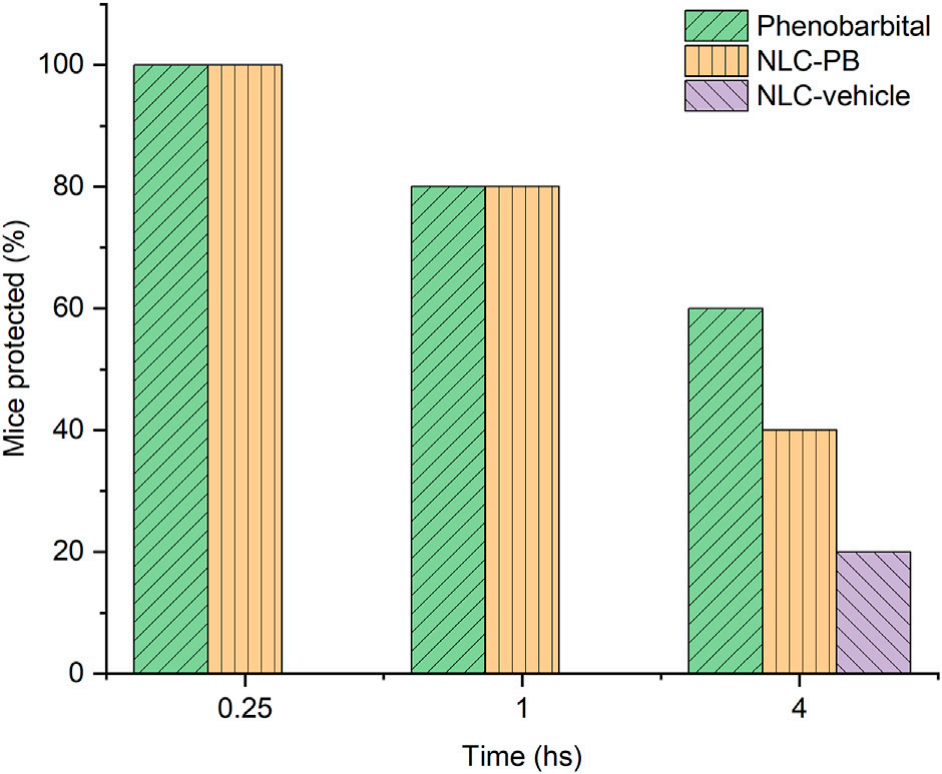
*In vivo* evaluation of the anticonvulsant activity expressed as proportion (%) of mice protected at each post-dose time. Green bars represent positive control (free PB), orange bars the optimized formulation (NLC-PB), and violet bars the vehicle without drug (NLC-vehicle). Both free PB and NLC-PB, were tested at a dose of 25 mg/kg.

The protection in the PTZ test for each treatment at the different time points after administration is shown in Figure 10. Results obtained for negative control (NS) were not included in the figure since all animals suffered from seizures after the administration of PTZ in all cases (i.e., 0% of protection at each time point). On the other hand, although the NLC-vehicle (violet bars) showed no anticonvulsant activity at the first time points, one mouse out of five tested, showed some activity at 4 h. Regarding PB, either as a free drug (green bars) or loaded into NPs (NLC-PB, orange bars), the protection against PTZ was similar between groups. It should be noted that the differences observed at 4 h, as well as the protection exerted by the NLC-vehicle, only represent 20%, that is, one animal out of five tested, which is within the inter-individual variability of the *in vivo* assay.

Additionally, none of the mice tested exhibited neurotoxic effects, after the administration of each treatment, when the RotoRod test was performed.

## 4 Discussion

Various strategies have been proposed to provide an effective solution for the treatment of refractory epilepsy, one of the most innovative being those focused on the design of pharmaceutical vehicles intended to deliver the drug to the site of action. In this sense, SLN and NLC have gained relevance due to their characteristics, similar to polymeric NPs, but with much less or no intrinsic toxicity, and greater stability. These systems have been demonstrated to be capable of penetrating the BBB (Martins et al., 2012; Graverini et al., 2018; Khonsari et al., 2022), improving drugs’ permeability through biological barriers and increasing their bioavailability (Masiiwa and Gadaga, 2018; He et al., 2019; Rana et al., 2020; Saini et al., 2021). This could be of special importance for drugs that, like in the case of PB, exhibit transport by ABC efflux transporters, which have been shown to be upregulated at the BBB of refractory patients (Aronica et al., 2012; Qosa et al., 2015). ABC transporters’ substrates encapsulated within nanocarriers could then mimic a Trojan horse strategy, hiding the drug molecules from direct interaction with efflux pumps at the BBB (Potschka, 2012).

When designing a new formulation, it is important to identify and characterize the variables that can modify its quality attributes and find the optimal conditions to achieve the desired (and previously established) properties or characteristics of the final product. Development based on a QbD approach is a key tool to achieving this goal (Cunha et al., 2020). In the present work, screening of variables or factors capable to affect three quality attributes (responses) were analyzed: size, Z-potential, and PDI. As expected, relevant factors were those related to the main components of the formulation (i.e., amount of lipid and surfactant), factors commonly identified as critical ones for lipid nanosystems (Cacicedo et al., 2019). The other variables studied (i.e., amount of oil, potency, and sonication time), were not statistically significant. Whereas in the case of the oil this result was probably due to its minor contribution to the formulation, potency, and sonication time levels used were enough to produce NPs with the desired size and PDI values, in all cases. Next, we study in more detail the effect of the two variables selected as relevant, to find the best formulation by simultaneously minimizing the three response variables.

The result in the NLC-PB optimized formulation, exhibited values of 178.6 nm, −12.2 mV, and 0.244 of particle size, Z-potential, and PDI, respectively. Moreover, the formulation achieved a drug encapsulation efficiency superior to 98%, and the dual stabilization system (steric and electric) was successful to provide more than 1 month of stability under storage conditions, with no perceptible aggregation of the particles. Normal refrigeration was selected as the storage condition since no antimicrobial agents were included in the formulation.

The size and morphology of the NPs were further studied by TEM and AFM, obtaining results consistent with those obtained by DLS and mentioned above. The observation of lipid NPs by AFM has, as main advantages, ease of sample preparation and the possibility of observing the particles in a hydrated state, similar to what occurs in suspension (Dubes et al., 2003). However, the AFM technique does not lack of disadvantages, which position it as a complementary or accessory technique in terms of determining the dimensions of lipid NPs. Due to the soft nature of these NPs, they tend to deform (flatten), due to attractive forces with the support, and the interaction with the probe (Dubes et al., 2003; Peixoto et al., 2005; Mazuryk et al., 2016). However, this technique allowed us to observe our system in a three-dimensional way which, together with the images obtained by TEM, made it possible to demonstrate the spheroidal morphology of the particles, as well as the absence of agglomerations.

DSC and TGA were employed to analyze the thermal properties of the optimized formulation. The thermograms indicate a good dispersion of the drug within the lipid matrix, i.e., the drug could be in an amorphous state or molecularly dispersed within the matrix, not showing the characteristic peak or thermal process of its crystalline state (Ruiz and Scioli Montoto, 2018). Whereas the DSC thermogram of NLC-PB presented the same characteristics as the NLC-vehicle. Besides, no characteristic peaks of PB could be observed in the thermograms. Also, the TGA analysis of the NLC-PB did not show a decomposition process that could be associated with PB, as it exhibited the same behavior as the vehicle. Moreover, XRD analysis is also indicative of a molecular dispersion of the drug in the NLC. As it can be seen from Table 5, both the vehicle and the NLC-PB XRD diffractogram presented four main peaks at 19.18°, 21.64°, 23.33°, and 23.90°. Whereas the peaks at 21.64° and 23.90° suggest the presence of a crystalline structure of the lipid matrix (particularly its polymorphic state β’), the peaks located at 19.18° and 23.33° would correspond to the Poloxamer 188 diffraction pattern, in agreement with the values found by other authors (Fathy and El-Badry, 2003). Therefore, it was not possible to identify any characteristic peak of PB in the NLC-PB formulation. The same conclusion can be reached from the FTIR results: NLC formulations, with or without PB, presented bands associated with myristyl myristate and poloxamer 188, but none of the PB characteristic bands could be observed in the NLC-PB sample spectrum. The small shift toward higher wavenumbers exhibited by some bands in the NLC formulations (for the individual components) is indicative of a partial restructuration of the lipid matrix after the incorporation of the surfactant (Rodenak-Kladniew et al., 2019).

The performance of the optimized formulation was tested in terms of its release behavior, cellular toxicity, and *in vivo* anticonvulsant effect. Optimized NLC-PB formulation exhibited a modified drug release rate in comparison with the conventional (immediate-release) tablet formulation. Although tablets and NLCs are intended for different administration routes, from a technological point of view this result means that the nanoformulation strategy was successful to provide a slower release of the drug from the matrix, which, in turn, may allow the maintenance of the therapeutic activity for an extended period, reducing toxic or adverse effects and/or targeting the drug release at specified time-points (EMA, 2014). Another interesting result of the release study was the lack of relationship between the fraction released and the initial amount of drug in the formulation and, thus, the good fit of the data to the proposed diffusion model. In line with the previously discussed thermal and spectroscopic results, this may indicate that the drug is not present as a solid or crystal but rather as a dispersion among the matrix components. If the drug was in the solid-state in the formulation, the dissolved fraction as a function of time would possibly be proportional, among other things, to the initial amount of drug in the formulation, a situation described by well-known models, such as Higuchi and Baker-Lonsdale structured models (Talevi and Ruiz, 2021).

Finally, the NLC-PB formulation did not exhibit toxicity against the assayed cell line [a result commonly found for lipid-based nanosystems, due to the non-toxic and biocompatible nature of the formulation ingredients (Rodenak-Kladniew et al., 2019; Andrade et al., 2020; Erel-Akbaba et al., 2021)] and was able to exert an anticonvulsant effect in the PTZ model of acute seizures (Figure 10). In terms of protection, the NLC-PB showed a behavior similar to the free drug. Therefore, we believe that this is a promising result since it indicates that the formulation was capable of delivering the drug to the site of action at the CNS in an adequate amount and time for its therapeutic effect, with no neurotoxic effects according to the RotoRod test. In the future, we consider studying other experimental designs that may reflect a differential behavior of the NPs to the free drug, such as an enhanced duration of the therapeutic effects (Cirri et al., 2017; Joseph et al., 2017) or an increased central bioavailability (Graverini et al., 2018; He et al., 2019) of the drug encapsulated into lipid NPs.

## 5 Conclusion

In the present work, phenobarbital was successfully loaded into rationally designed lipid-based nanovehicles. Physicochemical characterization showed that those NPs were spheroidal and with a mean particle size under 200 nm, physically stable over time. The DSC, TGA, XRD, and FTIR exhibited profiles compatibles with a molecular dispersion of the drug into the lipid matrix. *In vitro* release assay displayed a slower release rate when compared with a conventional, immediate-release commercial formulation. Neither PB, NLC-PB nor the empty NLC evidenced toxicity over the L929 cell line at the concentration range evaluated (from 1 to 4 mM). Finally, the *in vivo* anticonvulsant activity assay demonstrated a similar activity of NLC-PB to free PB. Therefore, these results suggest that NLC-PB might be an alternative drug delivery system to be used in the treatment of epilepsies.

## Data Availability

The raw data supporting the conclusion of this article will be made available by the authors, without undue reservation.
