# Supplementary material for: Novel Phenobarbital-Loaded Nanostructured Lipid Carriers for Epilepsy Treatment: From QbD to In Vivo Evaluation
Source: Front Chem. 2022 Aug 17;10:908386. doi: 10.3389/fchem.2022.908386 (PMC9428247; doi:10.3389/fchem.2022.908386)
Supplement: Supplementary file 1 [file DataSheet1.PDF]

Supplementary Material

Supplementary Table 1. Individual measurements of the three independent replicas of the optimized formulation (raw data from Table 3) used to validate de predictions obtained by RSM. For each sample (1, 2 and 3), particle size and PDI were measured 9 times, whereas Z-potential were measured 6 times, and the value of each sample were obtained by averaging repeated runs.

		**Size (nm)**		**PDI**		**Z-potential (mV)**	
**Sample**	**Run**	**Values**	**Mean**	**Values**	**Mean**	**Values**	**Mean**
**1**	1	181.6	180.1	0.245	0.250	-13.3	-12.6
	2	181.8		0.253		-11.4	
	3	180.6		0.255		-13.7	
	4	181.3		0.254		-11.3	
	5	169.5		0.242		-13.2	
	6	179.7		0.255		-12.9	
	7	181.8		0.250		---	
	8	181.7		0.249		---	
	9	183.2		0.248		---	
2	1	169.4	175.0	0.229	0.230	-11.0	-11.4
	2	173.1		0.244		-10.9	
	3	178.3		0.225		-10.6	
	4	174.6		0.231		-12.6	
	5	175.0		0.221		-12.0	
	6	177.4		0.236		-11.0	
	7	176.6		0.235		---	
	8	177.0		0.218		---	
	9	173.9		0.229		---	
3	1	168.5	180.6	0.239	0.252	-12.4	-12.5
	2	182.7		0.258		-12.4	
	3	186.2		0.266		-12.2	
	4	181.5		0.248		-12.1	
	5	181.6		0.268		-13.3	
	6	181.7		0.253		-12.5	
	7	182.8		0.257		---	
	8	181.9		0.241		---	
	9	178.1		0.240		---	
**Mean**			**178.6**		**0.244**		**-12.2**
**SD**			**3.1**		**0.012**		**0.7**

(A)

			Size (d.nm):	% Intensity:	St Dev (d.nm):
Z-Average (d.nm):	181,6	Peak 1:	208,3	100,0	81,96
PdI:	0,245	Peak 2:	0,000	0,0	0,000
Intercept:	0,947	Peak 3:	0,000	0,0	0,000
Result quality :	Good				

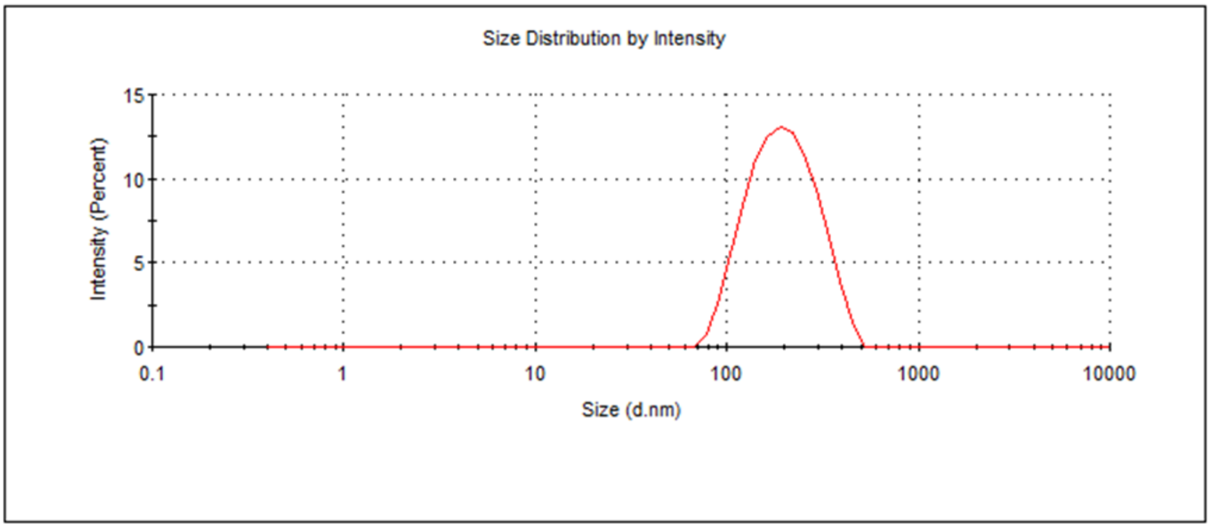

(B)

			Mean (mV)	Area (%)	St Dev (mV)
Zeta Potential (mV):	-13,3	Peak 1:	-13,3	100,0	18,8
Zeta Deviation (mV):	18,8	Peak 2:	0,00	0,0	0,00
Conductivity (mS/cm):	0,00265	Peak 3:	0,00	0,0	0,00
Result quality :	Good				

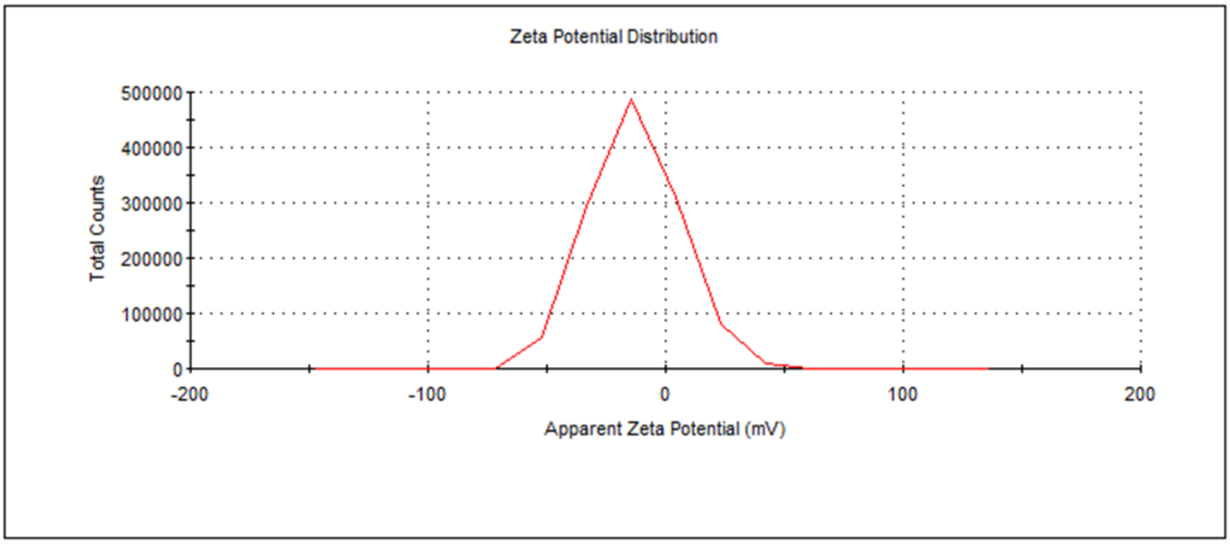

Supplementary Figure 2. Example of graphical outputs corresponding to the optimized formulation: (A) particle size and PDI; (B) Z-potential. Both graphs correspond to a single reading of one sample, in this case sample 1, run 1.
